# A case report of neuromyelitis optica spectrum disorder induced by pembrolizumab treatment for lung adenocarcinoma: a clinical and immunohistochemical study

**DOI:** 10.1186/s12883-022-02987-6

**Published:** 2022-12-15

**Authors:** Shigeki Hirano, Akira Kojima, Yoko Nakayama, Takahiro Takeda, Takashi Kishimoto, Toshiyuki Takahashi, Satoshi Kuwabara, Masahiro Mori

**Affiliations:** 1 Department of Neurology, Graduate School of Medicine, Chiba University, 1-8-1 Inohana, Chuo-ku, Chiba Chiba, 260-8670 Japan; 2 Department of Internal Medicine, Funabashi Central Hospital, Funabashi, Chiba Japan; 3 Department of Ophthalmology, Funabashi Central Hospital, Funabashi, Chiba Japan; 4 Department of Neurology, National Hospital Organization Chiba-Higashi Hospital, Chiba, Chiba Japan; 5 Department of Molecular Pathology, Graduate School of Medicine, Chiba University, Chiba, Chiba Japan; 6 Department of Neurology, Tohoku University Graduate School of Medicine, Sendai, Miyagi Japan; 7 Department of Neurology, National Hospital Organization Yonezawa National Hospital, Yonezawa, Yamagata Japan

**Keywords:** Neuromyelitis optica spectrum disorders, Immune checkpoint inhibitor, Pembrolizumab, Case report, Aquaporin-4

## Abstract

**Background:**

We report a case of neuromyelitis optica spectrum disorders (NMOSD), who developed after the pembrolizumab treatment, an immune checkpoint inhibitor, against lung adenocarcinoma. The present case is discussed with the lung adenocarcinoma specimen which was stained by aquaporin-4 (AQP4) and with literature review of NMOSD linked to immune checkpoint inhibitors.

**Case presentation:**

A 62-year-old Japanese man presented with acute diencephalic syndrome, left optic neuritis, and myelitis 5 months after initiation of pembrolizumab treatment for lung adenocarcinoma. He was diagnosed with NMOSD based on serum anti-aquaporin-4 (AQP4) antibody positivity. Immunohistochemistry of lung biopsy samples showed AQP4 expression on CD68+ cells. This is the fifth reported case of AQP4+ NMOSD triggered by an immune checkpoint inhibitor and the first with a brain lesion. Four out of five NMOSD cases, including the present case and one case with lung metastasis, had lung cancer.

**Conclusions:**

Immune checkpoint inhibitors may trigger AQP4+ NMOSD owing to their molecular similarity to AQP4 expressed in lung and glial tissues. Prompt brain/spinal cord imaging and anti-AQP4 antibody testing may facilitate early diagnosis of immune-mediated adverse event in central nervous system associated with immune checkpoint inhibitors.

## Introduction

Pembrolizumab, an inhibitor of programmed death 1 (PD-1), has applications in a wide range of advanced-stage malignancies, including non-small cell lung cancer (NSCLC). Pembrolizumab treatment enhances immunity against tumors and improves survival. It has recently replaced cytotoxic chemotherapy as a first-line therapy against advanced NSCLC lacking a driver mutation [1]. However, PD-1 inhibitor treatment may induce immune-mediated adverse events, including neurological events such as headache, encephalopathy, meningitis, Guillain-Barré-like syndrome, myasthenic syndrome, and central nervous system demyelinating disorders [2].

Neuromyelitis optica spectrum disorders (NMOSD) is an inflammatory central nervous system demyelinating syndrome, which is usually associated with serum anti-aquaporin-4 (AQP4) antibody (AQP4+ NMOSD) [3]. NMOSD core symptoms include optic neuritis, acute myelitis, area postrema syndrome, acute brainstem syndrome, symptomatic narcolepsy or acute diencephalic clinical syndrome, and symptomatic cerebral syndrome. Herein, we report a case of AQP4+ NMOSD induced by pembrolizumab treatment and compare it with previous reported cases.

## Case presentation

A 62-year-old right-handed Japanese man with type 2 diabetes mellitus presented with an 8-month history of hoarseness. Chest computed tomography revealed a space-occupying lesion in the hilum and apex of the left lung, which extended into the upper mediastinum (64 mm in short-axis diameter). Aspiration biopsy confirmed the diagnosis of lung adenocarcinoma. Immunohistochemistry revealed that the lesion was thyroid transcription factor 1 (TTF-1) positive, napsin A equivocal, and p40 negative.

Brain magnetic resonance imaging (MRI) before chemotherapy initiation did not reveal any brain metastasis or other abnormality. The tumor was graded as T4N3M0, stage IIIC. Molecular profiling of the *EGFR*, *ALK*, *ROS-1* genes revealed no sensitizing alterations. The anti-programmed death ligand 1 (PD-L1) tumor proportion score was < 1%.

Five months prior to neurological symptom onset, treatment with cisplatin 94 mg, pemetrexed 835 mg, and pembrolizumab 200 mg was initiated and repeated every 3 weeks for four courses, followed by four courses of pemetrexed 835 mg and pembrolizumab 200 mg treatment.

**Fig. 1:**
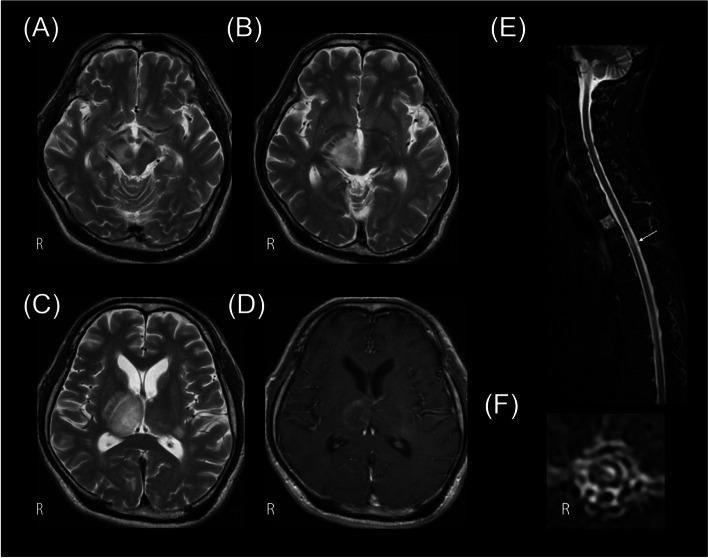
Transaxial brain magnetic resonance imaging (MRI) 1 month after neurological onset, showing T2 high intensity lesion in (**A**) the right cerebral peduncle, (**B**) diencephalon to (**C**) thalamus, adjacent to the third ventricle with (**D**) gadolinium enhancement. Spinal cord MRI 2 months after neurological onset showing short inversion recovery (STIR) images on (**E**) sagittal and (**F**) transaxial plane with bright spotty lesion at T3 level

At the last pemetrexed and pembrolizumab treatment, the patient experienced difficulty walking and became wheelchair bound within 2 weeks. He developed somnolence, left hemiplegic ataxia, dysarthria, and dysesthesia in the right arm. Ophthalmological examination showed a visual acuity of 0.2 in the right eye and 0.3 in the left eye, left-side predominant bilateral upper gaze, and left abduction and down gaze palsy. Brain MRI showed a T2 high-signal lesion adjacent to the ventricle in the right thalamus and the right posterior limb of the internal capsule and the right cerebral peduncle, with edema and gadolinium enhancement (Fig. 1A–D). Cerebrospinal fluid examination revealed 7 cells/μL, protein 109 mg/dL, and glucose 130 mg/dL. Serum anti-Hu, Ri, CRMP5, Ma2, amphiphysin, VGKC, AQP4, and myelin oligodendrocyte glycoprotein (MOG) antibodies were tested before steroid therapy initiation, of which only anti-AQP4 antibody tested positive in a cell-based assay [4], which confirmed the diagnosis of NMOSD. Written consent was obtained. Spinal MRI showed a left anterior intraspinal cord bright spotty lesion at the T3 level, extending for the length of two vertebral segments (Fig. 1E, F).

**Fig. 2:**
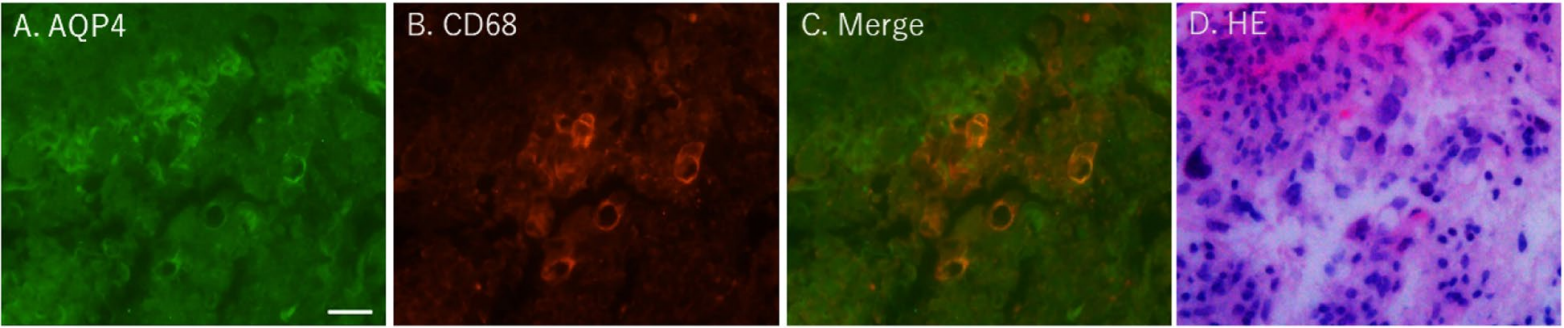
Immunohistochemical staining of lung adenocarcinoma. **A** Aquaporin-4 (AQP-4), (**B**) CD68, (**C**) AQP4 + CD68, (**D**) hematoxylin and eosin (H-E) stain. The biopsied specimen was fixed in 10% buffered formalin. After embedding the tissue in paraffin, 3–4-μm-thick sections were prepared. Immunohistochemical study was performed using antibodies against AQP-4 (4/18: sc-32,739, monoclonal, 1:100: Santa Cruz Biotechnology, Dallas, TX) and CD68 (bs-0649R, polyclonal, 1:500: Thermo Fisher Scientific, Waltham, MA). After digital recording of the fluorescent signals, the coverslips were removed, and the same slide was subjected to H-E staining to yield the bright-field counterpart of the fluorescence images, similarly recorded by the same equipment. The scale bar represents 20 μm. Some CD68-positive macrophages were positive for the AQP-4 staining

Immunohistochemical staining of the lung biopsy sample showed that AQP4 and cluster of differentiation (CD)68 were co-expressed on cells in the adenocarcinoma lesion, indicating that macrophages inside the tissue expressed the AQP4 antigen (Fig. 2).

Chemotherapy, including pembrolizumab, was discontinued. Dexamethasone 6.6 mg/day treatment was initiated, maintained, and tapered. The gadolinium-enhanced lesion disappeared, leaving a necrotic focus in the right thalamus. Left critical flicker fusion frequency was diminished (37 Hz) and left central visual field sensitivity transiently decreased, with full recovery. Four months after the onset of neurological signs, the patient’s consciousness was clear with normal sensation, but his left eye palsy remained. He was able to stand alone and walk a few meters with assistance.

## Discussions and conclusion

The diagnosis of NMOSD by the 2015 International Panel on neuromyelitis optica Diagnosis (IPND) was defined as, AQP4 antibody seropositive patients with only a single compatible syndrome (optic nerve, spinal cord, area postrema, other brainstem, diencephalic, or cerebral presentations) or AQP4 antibody seronegative patients, dissemination in space is necessary in addition to certain MRI requirements [3]. To the best of our knowledge, four cases of AQP4+ NMOSD and a case of seronegative NMOSD induced by PD-1 inhibitor treatment have been reported until date (Table 1) [5–9]. Four of the six patients had lung cancer (including one lung metastasis), there were one case each of Hodgkin lymphoma, clear cell kidney cell carcinoma, and uveal melanoma. Four cases were induced by nivolumab treatment and two were induced by pembrolizumab. Their NMOSD symptoms started 2 weeks to 11 months after starting ICI treatment. Four patients presented with myelitis, one with optic neuritis, the present case being the first to present with brain lesion. Five patients showed partial recovery and one died.

**Table 1:** Summary of prior reported and present cases of neuromyelitis optica spectrum disorders induced by immune checkpoint inhibitors

First author, publication year [reference]	Narumi Y, 2018 [5]	Shimada T, 2020 [6]	Nasralla S, 2020 [7]	Weiss D, 2021 [8]	Khimani K, 2022 [9]	Present case
**Age (years), sex**	75, male	63, female	30, female	81, female	57, male	62, male
**Country**	Japan	Japan	USA	Germany	USA	Japan
**Primary cancer**	Lung squamous cell carcinoma	Lung adenocarcinoma	Hodgkin lymphoma	Clear cell renal cell carcinoma, lung metastasis	Uveal melanoma	Lung adenocarcinoma
**ICI (number of cycles)**	Nivolumab (1)	Pembrolizumab (1)	Nivolumab (3)	Nivolumab (10)	Nivolumab (9) Ipilimumab (3)	Pembrolizumab (8)
**Clinical subtype**	Myelitis	Myelitis	Myelitis, optic neuritis	Myelitis	Optic neuritis	Diencephalic, myelitis, optic neuritis
**Onset from the onset of ICI**	2 months	2 weeks	8 weeks	11 months	7 months	5 months
**Cerebrospinal fluid**	Cells 1195/μL, protein 380.9 mg/dL	Cells 9/μL, protein 48 mg/dL	Cells 345/μL, protein 516 mg/dL	Cell 65/μL protein 63 mg/dL	Not done	Cells 7/μL, protein 109 mg/dL
**Anti-AQP4 antibody**	+	+	–	+	+	+
**Anti-MOG antibody**	–	–	–	–	–	–
**Treatment**	Discontinued ICI, steroid pulse therapy, plasmapheresis	Discontinued ICI, IV methylprednisolone, oral prednisone, plasma exchange	Discontinued ICI, IV methylprednisolone, oral prednisone, rituximab	Discontinue ICI, iv methylprednisolone, plasma exchange	Discontinued ICI, IV methylprednisolone, oral prednisone, rituximab	Discontinued ICI, IV dexamethasone
**Outcome**	Partial recovery	Partial recovery	Partial recovery	Died	Partial recovery	Partial recovery

*Abbreviations*: *ICI* Immune checkpoint inhibitor, *IV* Intravenous

A meta-analysis of central nervous system demyelinating disorders associated with immune checkpoint inhibitor (ICI) therapy published in 2018 revealed 23 cases, including seven of myelitis, five of multiple sclerosis, four of isolated optic neuritis, one of NMOSD, [5] and six of atypical demyelination [2]. Nine cases of optic neuritis have been reported as adverse events after ICI induction [10].

The PD-1/PD-L1 pathway can inhibit self-reactive T cells and protect against autoimmunity via induction of regulatory T cells (Tregs) and direct inhibition of pathogenic self-reactive T cells. T-cell responses to AQP4 and Th17 deviation with decreased Treg count have been reported in patients with AQP4+ NMOSD [11]. T-cell responses may play a role in the development of AQP4+ NMOSD after ICI.

Immunohistochemistry of the lung biopsy samples suggested that lung AQP4 antigen was scavenged by local CD68-positive macrophages, which may lead to antigen (AQP4) presentation. Notably, four of the five reported cases, including the present case, of AQP4+ NMOSD combined with neoplasm were associated with lung cancer [5, 6, 8]. Additionally, paraneoplastic NMOSD expressing AQP4 has been reported in a patient with lung adenocarcinoma [12]. The amount of AQP4 in lung adenocarcinoma has been reported to be not significantly different [13] or even less [14] than the normal lung tissue. Therefore, it is not the amount of AQP4 on the tissue from lung cancer but the production of anti-AQP4 antibody induced by ICI may be critical for the patogenesis of NMOSD with lung cancer treated with ICI. ICI may trigger AQP4+ NMOSD owing to their molecular similarity to AQP4 expressed in lung and glial tissues.

It is unknown whether our patient wasanti-AQP4 antibody positive before chemotherapy initiation. This should be addressed in future studies. In this case, it is not possible to rule out the probability of a paraneoplastic syndrome, but the clinical course suggested that ICI treatment played an important role in disease induction.

In conclusion, we reported a patient with NMOSD who developed diencephalon clinical syndrome and optic neuritis with myelitis after pembrolizumab treatment for lung adenocarcinoma. This is the fifth reported case of AQP4+ NMOSD induced by treatment with a PD-1 inhibitor and the first with encephalitis. Although AQP4+ NMOSD as an immune-mediated adverse event of PD-1 inhibitor is rare, prompt brain/spinal cord imaging and anti-AQP4 antibody testing may facilitate early diagnosis.

## Data Availability

The datasets used and/or analysed during the current study are presented within the manuscript. Although, data may be available from the corresponding author on reasonable request.

## Abbreviations

AQP4: Aquaporin-4
ICI: Immune checkpoint inhibitor
NMOSD: Neuromyelitis optica spectrum disorders
NSCLC: Non-small cell lung cancer
PD-1: Programmed death-1
PD-L1: Programmed death ligand-1
